# Concordant Changes of Plasma and Kidney MicroRNA in the Early Stages of Acute Kidney Injury: Time Course in a Mouse Model of Bilateral Renal Ischemia-Reperfusion

**DOI:** 10.1371/journal.pone.0093297

**Published:** 2014-04-02

**Authors:** Melissa A. Bellinger, James S. Bean, Melissa A. Rader, Kathleen M. Heinz-Taheny, Jairo S. Nunes, Joseph V. Haas, Laura F. Michael, Mark D. Rekhter

**Affiliations:** 1 Cardiometabolic Diseases and Diabetic Complications, Lilly Research Laboratories, Eli Lilly and Company, Indianapolis, Indiana, United States of America; 2 Investigational Pathology, Lilly Research Laboratories, Eli Lilly and Company, Indianapolis, Indiana, United States of America; 3 Covance Laboratories, Greenfield, Indiana, United States of America; 4 Statistics-Discovery/Development, Lilly Research Laboratories, Eli Lilly and Company, Indianapolis, Indiana, United States of America; University of Florence, Italy

## Abstract

**Background:**

Acute kidney injury (AKI) is a syndrome characterized by the rapid loss of the kidney excretory function and is strongly associated with increased early and long-term patient morbidity and mortality. Early diagnosis of AKI is challenging; therefore we profiled plasma microRNA in an effort to identify potential diagnostic circulating markers of renal failure. The goal of the present study was to investigate the dynamic relationship of circulating and renal microRNA profiles within the first 24 hours after bilateral ischemia-reperfusion kidney injury in mice.

**Methodology/Principal Findings:**

Bilateral renal ischemia was induced in C57Bl/6 mice (n = 10 per group) by clamping the renal pedicle for 27 min. Ischemia-reperfusion caused highly reproducible, progressive, concordant elevation of miR-714, miR-1188, miR-1897-3p, miR-877*, and miR-1224 in plasma and kidneys at 3, 6 and 24 hours after acute kidney injury compared to the sham-operated mice (n = 5). These dynamics correlated with histologic findings of kidney injury and with a conventional plasma marker of renal dysfunction (creatinine). Pathway analysis revealed close association between miR-1897-3p and Nucks1 gene expression, which putative downstream targets include genes linked to renal injury, inflammation and apoptosis.

**Conclusions/Significance:**

Systematic profiling of renal and plasma microRNAs in the early stages of experimental AKI provides the first step in advancing circulating microRNAs to the level of promising novel biomarkers.

## Introduction

Acute kidney injury (AKI) is a syndrome characterized by the rapid loss of the kidney excretory function [1]. It is strongly associated with increased early and long-term patient morbidity and mortality, as well as subsequent development of chronic kidney disease [2]. AKI is typically diagnosed by the accumulation of end products of nitrogen metabolism (urea or creatinine) and/or decreased urine output [1]. Although these traditional markers are reliable, they lack the sensitivity and specificity that are necessary for early diagnosis. Multiple novel protein markers of AKI have been proposed and tested. However, early diagnosis of AKI in clinical conditions by using new serum and urinary biomarkers remains cumbersome [3].

Challenges for developing new protein-based biomarkers include the complexity of protein composition in blood, diversity of post-translational modifications, low relative abundance of many proteins of interest, and the difficulties in developing suitable high-affinity detection agents [4]. Circulating microRNA might provide a reasonable alternative. MicroRNAs are recognized as crucial regulators of gene expression. MicroRNAs are short non-coding RNAs that regulate gene expression by binding to specific mRNA targets and promoting their degradation and/or translational inhibition [5]. Importantly, microRNAs have been found in plasma and urine [6]. They are stable in various bodily fluids, the sequences of most microRNAs are conserved among different species, expression of some microRNAs is specific to tissues, and the level of microRNAs can be assessed by various methods, including those which allow for signal amplification [4].

Indeed, circulating miR-210 predicted improved rates of survival in patients with AKI [7]; whereas plasma levels of miR-21 were associated with progression of AKI [8] and levels of miR-494 were dramatically increased in the urine of AKI patients [9]. However, it is difficult to gain comprehensive understanding of a potential marker solely based on the clinical studies. One missing link is a systematic analysis of relationships between renal and plasma or urine microRNAs. Critical mass of studies done in various animal models of AKI is needed. To the best of our knowledge, only a few published studies to date have attempted this kind of analysis [10] [11]. Also, the majority of studies in animal models focus on relatively late time points when traditional markers are already substantially elevated or even on the decline, potentially reflecting initiation of tissue regeneration.

The goal of the present study was to investigate the dynamics of circulating and renal microRNA profile within the first 24 hours after bilateral ischemia-reperfusion kidney injury in mice. We have demonstrated highly reproducible, progressive, and concordant elevation of miR-714, miR-1188, miR-1897-3p, miR-877*, and miR-1224 in plasma and kidneys at 3, 6 and 24 hour after acute kidney injury. These dynamics correlated with histologic findings of kidney injury and with an established plasma marker of renal dysfunction (creatinine).

## Methods

### Animals

The investigation conforms to the *Guide for the Care and Use of Laboratory Animals* published by the US National Institutes of Health (NIH Publication No. 85-23, revised 1996). Experimental procedures using animals were approved by the Eli Lilly Institutional Animal Care and Use Committee. C57B6 mice were obtained from Harlan Laboratories and maintained on normal diet.

At the age of 9 weeks, mice were anesthetized with ketamine/xylazine and were subjected to renal ischemia-reperfusion. To induce ischemia, renal pedicles were approached via two flank incisions and clamped for 27 minutes with microaneurysm clamps. During the period of ischemia, body temperature was maintained by placing the mice on a 37°C heating pad and using a heat lamp. After removal of the clamps, the kidneys were inspected for restoration of blood flow. The skin was closed with metal clips. Mice were euthanized at 3, 6, and 24 hours after surgery. Control animals underwent sham surgical procedure and were euthanized at the same time points post surgery. For each time point, 5 sham-operated mice and 10 mice that underwent ischemia-reperfusion were used.

At the end of study, mice were euthanized by carbon dioxide asphyxiation followed by exsanguination by cardiac puncture. Blood samples were collected via cardiac puncture using Vacutainer tubes containing EDTA. The right kidney was frozen in liquid nitrogen for RNA analysis while the left kidney was fixed in 10% neutral buffered formalin for histology.

### Histology

Formalin-fixed kidneys were transversely trimmed, routinely processed, paraffin-embedded, microtomed at a thickness of 5 μm, and stained with hematoxylin and eosin. Tissue sections were examined by light microscopy and graded for tubular degeneration/necrosis. For tubular vacuolation, grading was based on intensity of vacuolation rather than percentage of tubules affected since most tubules in the cortex contained some degree of vacuolation regardless of the grade. In order to evaluate compound effects compared to controls, each severity grade for tubular degeneration/necrosis and tubular vacuolation was given a score that ranged from 0 to 5 (normal to severe). Specific criteria are delineated below. Within normal limits (Score 0): no changes, or changes consistent with spontaneous background finding in the age, gender, and/or strain; minimal (Score 1): 0–10% of the tubules in the kidney section were affected; slight (score 2): 10% to 25% of the tubules in the kidney section were affected; moderate (Score 3): 25% to 50% of the tubules in the kidney section were affected; marked (Score 4): 50% to 75% of the tubules in the kidney section were affected; severe (Score 5): >75% of the tubules in the kidney section were affected.

### Creatinine

EDTA blood was centrifuged at 14,000×g for 10 min to obtain plasma. Creatinine was analyzed by enzymatic method using a Roche Hitachi Modular Chemistry analyzer.

### MicroRNA Profiling

Right kidneys were homogenized while still frozen using a Tissuemiser (Fisher, PA). 35 mg of the homogenate was used in subsequent isolation steps. Total RNA (including microRNA) was isolated from kidneys using miRNeasy kit (Qiagen, CA) according to manufacturer’s protocol. Kidney RNA concentration and quality was assessed using the NanoDrop 1000 and the 2100 Bioanalyzer (Agilent, CA).

Total RNA (including microRNA) was isolated from EDTA plasma (∼100 ul) using miRNeasy Serum/Plasma kit (Qiagen) according to manufacturer’s protocol. C. elegans miR-39 miRNA mimic was spiked in at the beginning of isolation procedure for normalization purposes.

Equal volumes (plasma) or amount (kidney) of RNA were pooled within each group. Mature microRNA was reverse transcribed using miScript II RT kit (Qiagen) according to manufacturer’s protocol. RT PCR was performed on the cDNA using Mouse miRNome miScript miRNA PCR Arrays (v16.0 Qiagen) on the ABI 7900HT (Life Technologies, NY) in accordance with the manufacturer’s protocol. Fold changes were calculated using the delta delta CT method using C. elegans miR-39 as housekeeping gene for plasma. The average of all housekeeping genes on array (SNORD61, SNORD68, SNORD72, SNORD95, SNORD96A, and RNU6-2) were used for kidneys. Each timepoint’s Sham group was used as the control group. In short, the CT value of housekeeping gene was subtracted from CT value of gene of interest to obtain the delta CT value. Then the delta delta CT value was calculated by subtracting the delta CT value of the untreated sample (control) from the delta CT value of each test sample. Finally, fold change was calculated by taking the log base 2 of the negative delta delta CT value.

Select microRNAs were chosen for confirmation on individual animals. 2 ul of plasma RNA and 1000 ng kidney RNA were reverse transcribed as described above. RT PCR was performed on the kidney and plasma cDNA using mouse miScript primer assays (Qiagen). Fold changes were calculated as described above. C. elegans miR-39 and SNORD61 were used as housekeeping genes for plasma and kidneys respectively.

### mRNA Analysis

Total RNA (1000 ng) from kidneys was reverse transcribed with High Capacity cDNA Reverse Transcription kit (Life Technologies) using a Peltier Thermal Cycler (MJ Research, Canada). Thermocycler settings were 25°C for 10 min, 37°C for 2 hrs, then 85°C for 5 min. RT PCR was performed on the kidney cDNA using Assays on Demand primer/probesets (Life Technologies). PCR parameters were as follows: Stage 1 = 50°C for 2 min; Stage 2 = 95°C for 10 min; Stage 3 = 95°C for 15 sec then 60°C for 1 min (40 cycles). Fold changes were calculated as described above. 36b4 primer/probeset from Operon, AL (forward primer = ggcccgagaagacctcctt, reverse primer = tcaatggtgcctctggagatt, probe = [6-Fam]ccaggctttgggcatcaccacg[TamraQ]) was used as housekeeping gene to normalize.

### Statistics

The two-sample t-test was performed on log-transformed data to assess microRNA fold changes over sham within each time point. The three p-values were adjusted by the Bonferroni method (i.e., p-values were multiplied by three) for each microRNA [12]. Correlation was performed using Spearman method [12] on the log transformed values of creatinine and the log transformed values of microRNA fold changes. All the analyses were performed using JMP 8.0 software.

## Results

### Histology and Creatinine

**Figure 1:**
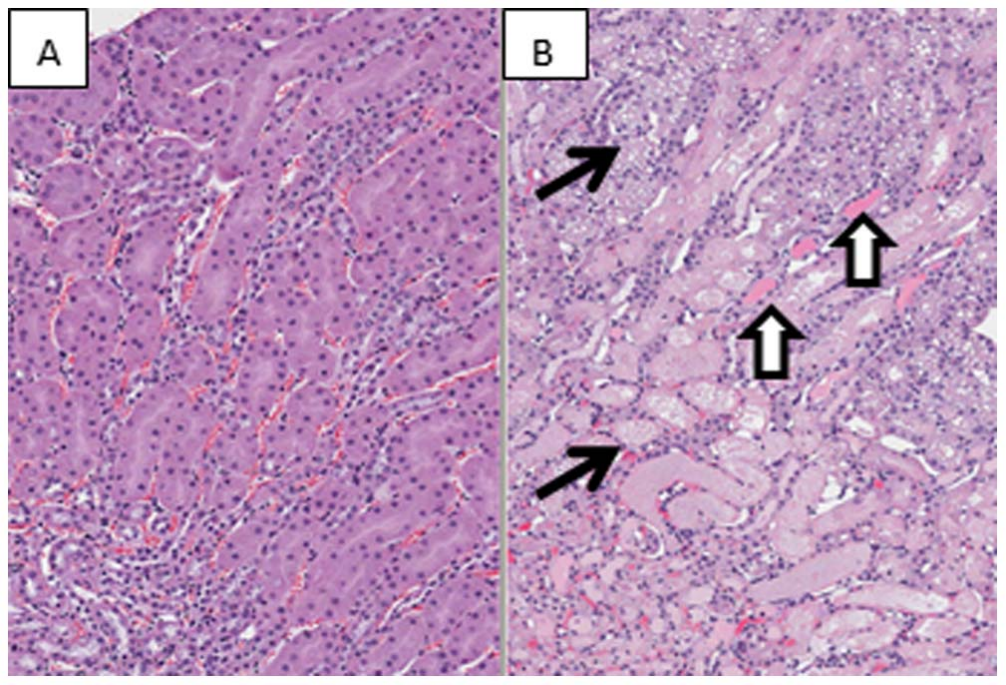
Histopathology of mouse kidneys associated with ischemia-reperfusion. **A**, sham operation. **B**, Twenty four hours after ischemia-reperfusion. Coagulative necrosis of the proximal tubule (arrows). Neutrophils have been recruited to the interstitium adjacent to areas of necrosis. Protein casts are scattered throughout the section (block arrows). Hematoxylin and Eosin, 100× magnification.

**Figure 2:**
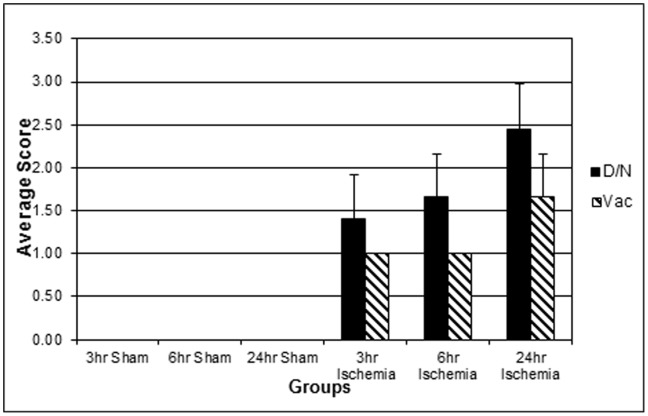
Distribution of average histopathology score. Kidney sections from the mice that underwent sham operation or bi-lateral renal ischemia –reperfusion were stained with hematoxilin-eosin (as represented in Figure 1). These sections were evaluated in a semi-quantitative manner for the signs of tissue injury. Degeneration, necrosis and vacuolation were not detected in the sham-operated animals while they increased in the kidneys with ischemia-reperfusion. Morphological signs of kidney injury progressively accumulated in accordance with reperfusion time. D/N = degeneration/necrosis score; Vac = vacuolation score.

No histologic findings were observed in sham operated and sacrificed at different times after sham surgery mice. Progressive tubular degeneration and necrosis were observed with increased time of sampling after ischemia (Figures 1 and 2). Tubular degeneration/necrosis was characterized by acute loss, fragmentation and/or attenuation of tubular epithelium centered on the corticomedullary junction, consistent with ischemia/reperfusion of the renal vessels and their tributaries, the arcuate vessels (Figure 2). Most affected tubules were in the medulla (outer stripe and medullary rays) and adjacent cortex following the vasculature along the collecting ducts. Affected tubules were variably dilated and contained exfoliated, degenerating/necrotic cells and cellular and/or granular eosinophilic debris (casts), occasionally admixed with proteinaceous fluid. Tubular vacuolation was limited to cortical tubules not affected by the degeneration/necrosis, mainly in the proximal and distal convoluted tubules. Affected tubular epithelial cells contained several, 2–6 μm, clear, sharply defined vacuoles.

**Figure 3:**
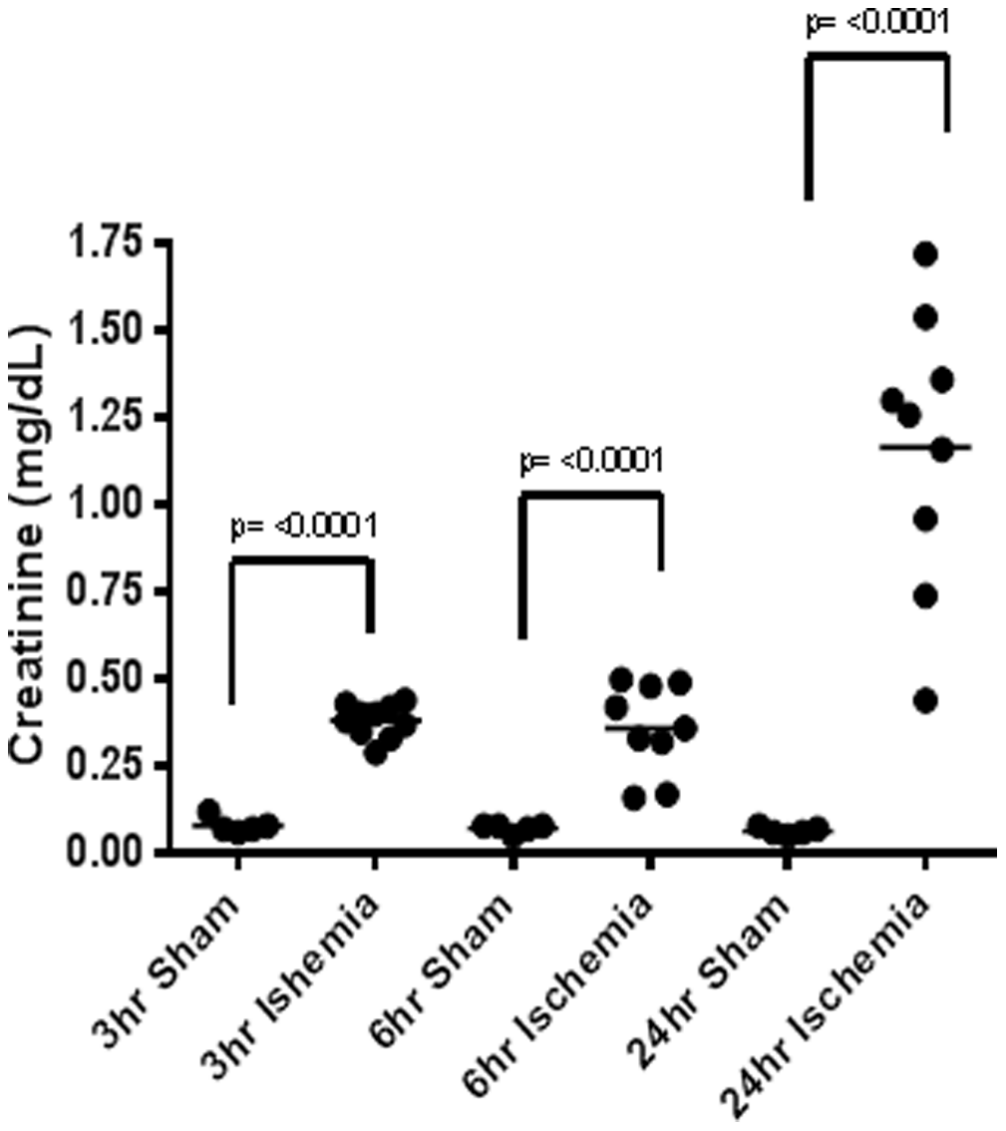
Changes of plasma creatinine in the time course of renal ischemia-reperfusion. Creatinine is a conventional circulating marker that is elevated when the kidney excretory function is impaired. In the sham-operated mice, plasma creatinine levels were at or below detection, while they were significantly increased in the animals that underwent bilateral renal ischemia-reperfusion. Plasma creatinine levels progressively grew over the time of reperfusion. Time points on the graph represent the time of reperfusion.

Plasma creatinine was significantly elevated (4.7 fold, p<0.0001) compared to sham as early as 3 hours after ischemia. By 24 hours, plasma creatinine levels rose 18 fold (p<0.0001) over sham-operated group (Figure 3).

### MicroRNA Array Results on Pooled Samples

Of the 940 microRNAs included on the kidney array, 57 showed a time dependent change (sustained throughout the three time points tested) with a maximum magnitude greater than 2 fold. One hundred eighteen microRNAs on the plasma array were modulated in the same manner. Eighteen microRNAs were modulated time dependently in both kidneys and plasma (Tables 1 and 2).

**Table 1:** MicroRNAs discordantly modulated in kidneys and plasma (pooled sample results).

miR	Kidney			Plasma		
	3 hr	6 hr	24 hr	3 hr	6 hr	24 hr
mmu-miR-467c	−2.11	1.13	2.40	4.01	3.05	1.71
mmu-miR-217	2.05	2.89	−1.17	2.15	3.15	6.51
mmu-miR-675-5p	3.78	−1.09	−2.05	−1.11	4.22	7.04
mmu-miR-1935	2.75	2.17	1.62	2.99	5.65	6.11
mmu-miR-1946a	2.34	2.08	1.40	−1.22	2.77	4.74
mmu-miR-669i	2.99	2.13	1.15	3.76	4.64	9.08
mmu-miR-709	2.97	1.50	1.30	3.81	11.05	13.56
mmu-miR-511-5p	2.66	2.01	1.11	−1.14	1.60	2.63
mmu-miR-2183	4.29	2.95	1.46	2.01	4.55	6.52
mmu-miR-1894-3p	10.09	1.84	−1.82	1.85	6.37	11.41
mmu-miR-696	3.93	3.42	1.02	3.46	6.18	24.85
mmu-miR-195*	3.54	2.13	1.17	2.13	3.67	7.02

**Table 2:** MicroRNAs concordantly modulated in kidneys and plasma (pooled sample results).

miR	Kidney			Plasma		
	3 hr	6 hr	24 hr	3 hr	6 hr	24 hr
mmu-miR-714	3.21	2.72	6.23	1.59	5.52	13.72
mmu-miR-1188	1.55	2.50	4.13	1.69	8.20	9.20
mmu-miR-1897-3p	3.72	5.82	11.12	7.88	4.12	13.12
mmu-miR-877*	1.38	2.28	2.90	1.91	2.21	5.84
mmu-miR-3471	3.77	2.59	1.06	11.04	10.25	10.10
mmu-miR-1224	1.45	1.83	4.77	−1.23	4.23	9.14

**Figure 4:**
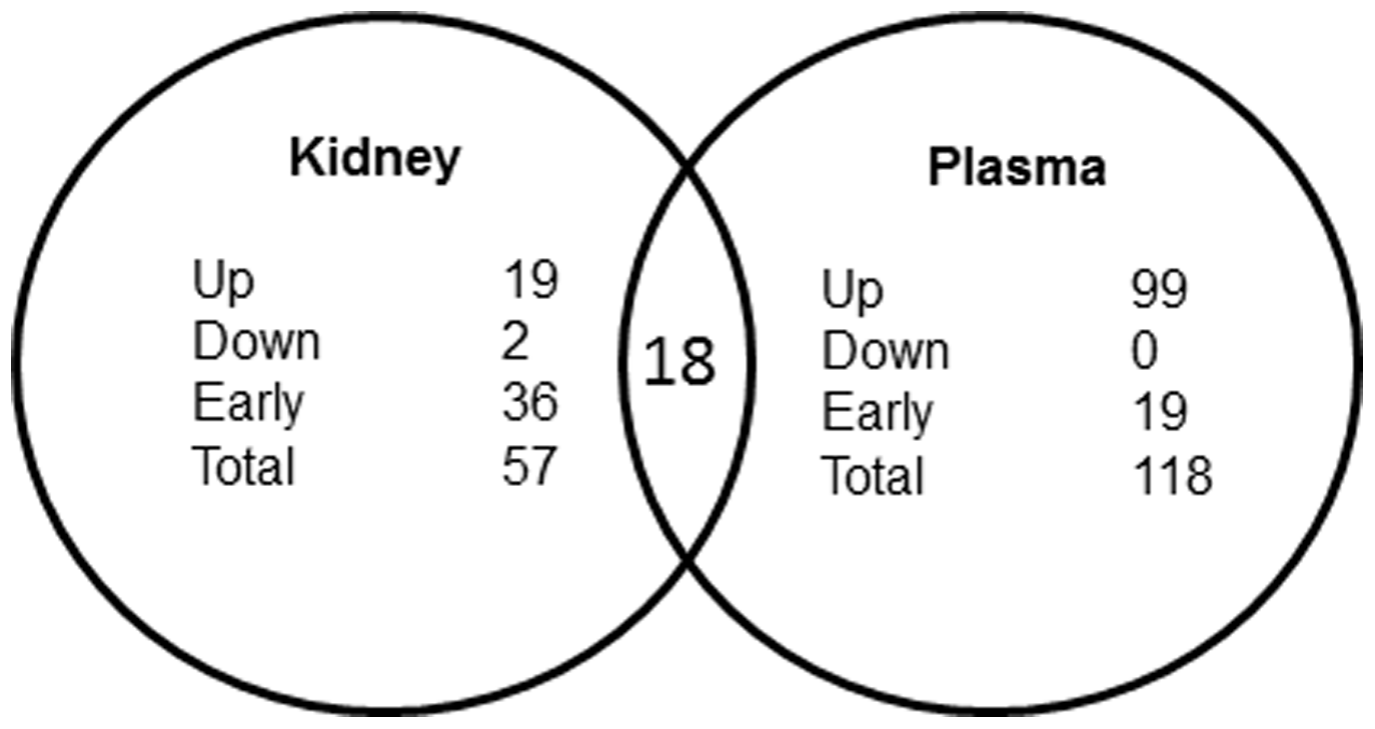
Pattern of microRNA modulation in kidneys and plasma of mice undergoing ischemia-reperfusion. While multiple microRNA species were modulated in the kidneys and in plasma after ischemia-reperfusion, 18 microRNAs were modulated in both compartments in a time-dependent manner. **Up**, increased expression; **Down**, decreased expression; **Early**, modulated at 3 hours and returned to the Sham level at 24 hours.

Of the 57 kidney microRNAs, 19 were increased in a time dependent fashion, 2 microRNAs were decreased, and 36 microRNAs were modulated at the 3 hour timepoint then returned towards sham levels at 24 hrs. Of the 118 plasma microRNAs, 99 were increasing time-dependently, none were decreasing, and 19 were modulated at 3 hours then returned towards sham levels at 24 hours. Of the 18 microRNAs that were time dependently modulated in both the kidneys and plasma, 10 were regulated in opposite directions while 6 were modulated in the same direction (Figure 4). In order to identify miRNAs likely arising from the kidney that could serve as plasma-borne biomarkers of renal injury, we focused our analysis on miRNAs that were regulated in both kidney and plasma.

### MicroRNA Confirmation on Individual Samples

**Figure 5:**
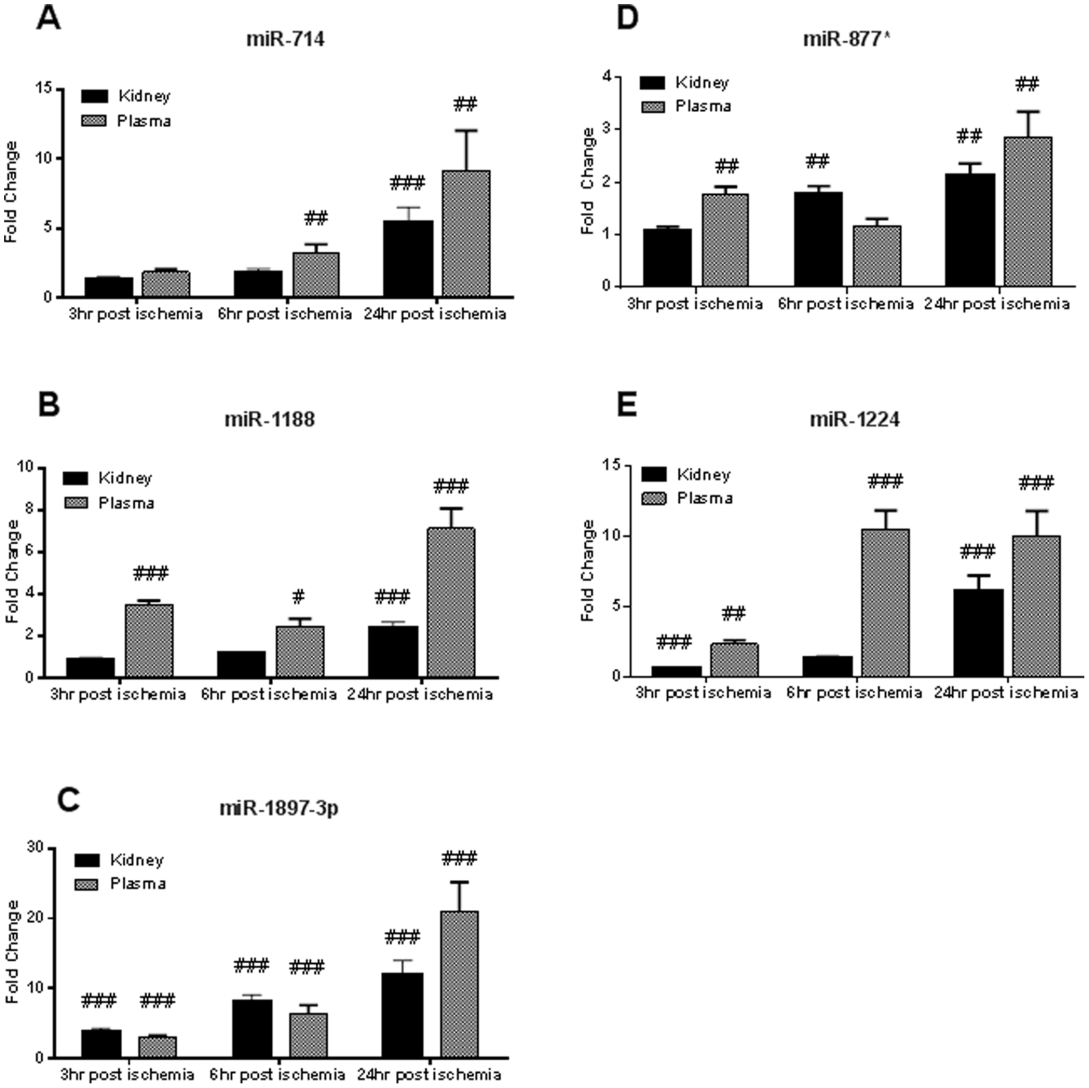
MicroRNA expression confirmation by real-time PCR in the samples from individual animals. Eleven microRNA species were selected on the basis of concordant and discordant changes in the kidney and plasma as detected in the pooled samples. The changes in five microRNAs were confirmed in the samples from individual animals. Bars on graphs represent fold changes from the time-matched sham-operated animals. MicroRNA expression levels form the sham-operated animals (not shown on graphs) are set to 1. Panel A shows miR-714 changes in kidney and plasma. Panel B shows miR-1188. Panel C shows miR-1897-3p. Panel D shows miR-877*. Panel E shows miR-1224. Two sample t tests were performed on sham and ischemic groups at each timepoint, then p values were adjusted with Bonferroni correction. # = p<0.05; ## = p<0.01; ### = p<0.001.

Eleven of these microRNAs (6 going in the same direction and 5 going in opposite directionswith the highest degree of modulation) were chosen for confirmation on individual animals. Of the eleven microRNAs retested, five (miR-1188, miR-1897-3p, miR-714, miR-877*, and miR-1224) confirmed in both kidney and plasma (Figure 5).

miR-1897-3p, miR-1188, miR-714, miR-877*, and miR-1224 increased time dependently in both kidneys and plasma. miR-1897-3p increased with the highest magnitude (12.07 fold in kidney, p<0.001; 20.96 fold in plasma, p<0.001) in both compartments and in the kidney it reached statistical significance the earliest (3.92 fold at 3 hours, p<0.001 ).

Importantly, miR-1897-3p, miR-1188, and miR-714 levels tightly correlated with plasma creatinine and with each other (Table 3). The observed co-regulation of miRNAs with the traditional marker of renal function–creatinine–indicated that these candidate miRNAs may be diagnostic markers or possibly mediators of the disease etiology.

**Table 3:** Correlation of plasma miR-1897-3p, miR-1188, and miR-714 with plasma creatinine.

Variable	By Variable	Spearman ρ	Prob> |ρ|
Log miR-1897-3p fold change	Log Creatinine	0.8427	<0.0001
Log miR-1188 fold change	Log Creatinine	0.8955	<0.0001
Log miR-1188 fold change	Log miR-1897-3p fold change	0.7820	<0.0001
Log miR-714 fold change	Log Creatinine	0.8858	<0.0001
Log miR-714 fold change	Log miR-1897-3p fold change	0.8183	<0.0001
Log miR-714 fold change	Log miR-1188 fold change	0.8096	<0.0001

### Pathway Analysis and Target Confirmation

**Figure 6:**
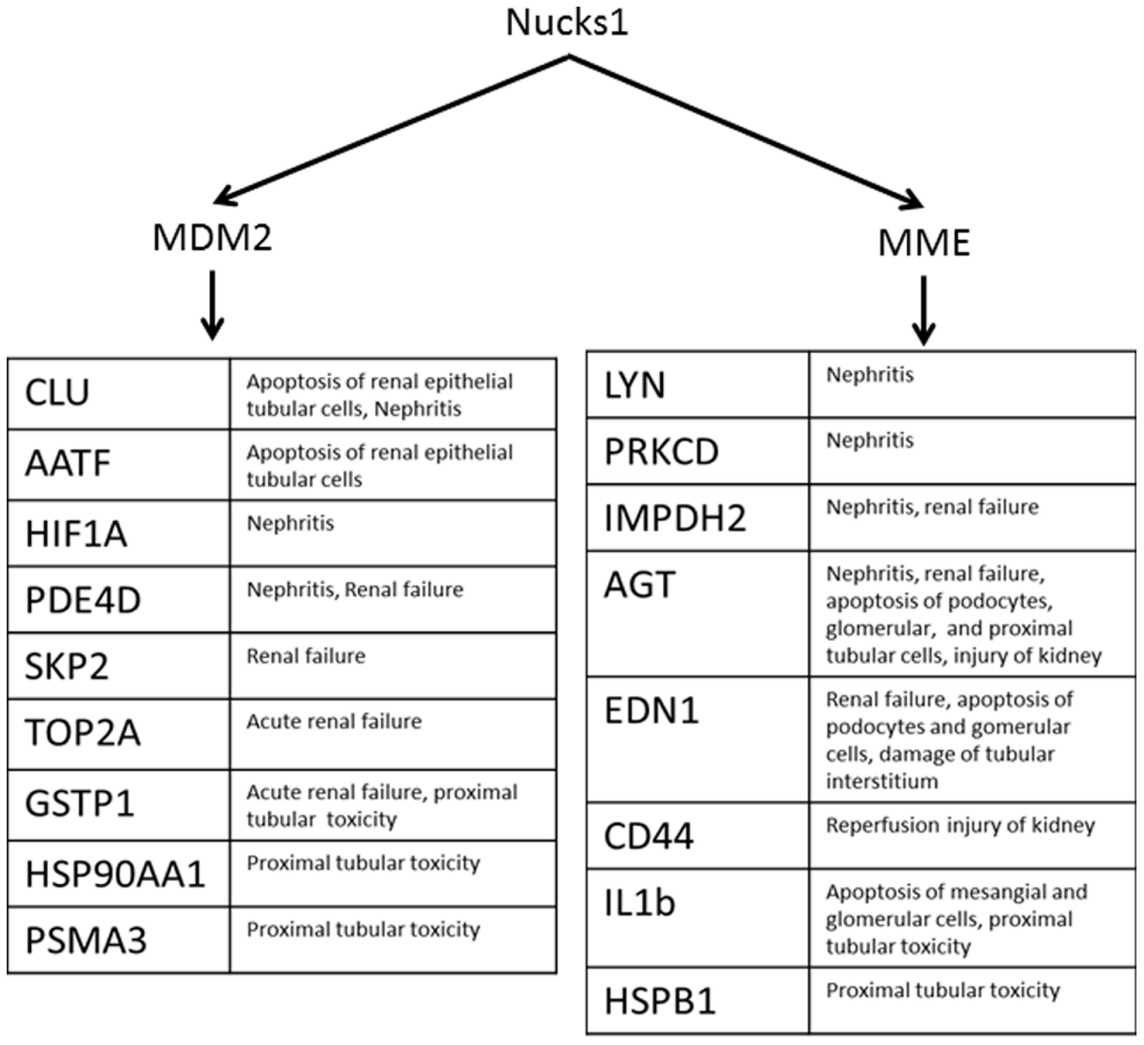
Downstream targets of Nucks1. Pathway analysis was performed on Nucks1 (nuclear casein kinase and cyclin-dependent kinase substrate 1) using Ingenuity Pathway Analysis. Downstream targets of Nucks1 appear to be involved in renal injury, inflammation, and apoptosis.

**Figure 7:**
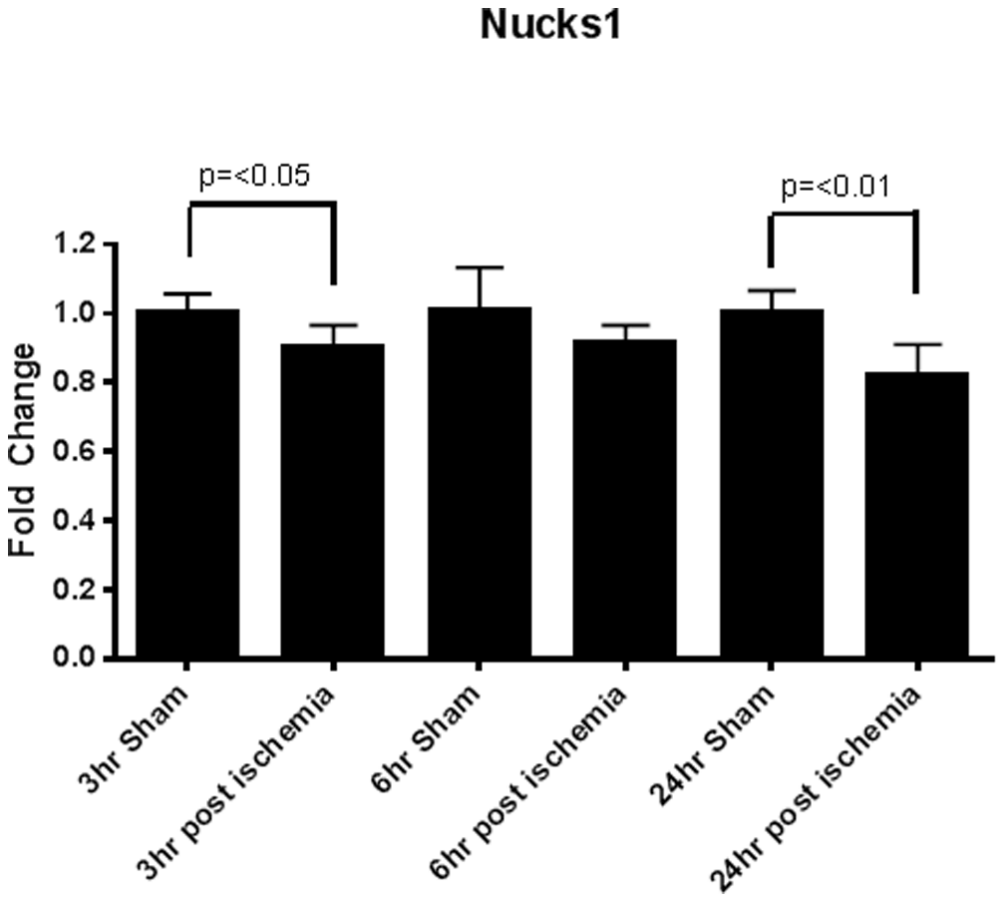
Nucks1 expression in the kidneys of mice with renal ischemia-reperfusion. Nucks1 gene expression was down-regulated in the injured kidneys at all time points and reached significance at 3 and 24 hours after reperfusion.

Because miR-1897-3p had the greatest modulation, our goal was to identify its mRNA targets and evaluate possible regulation of pathogenic pathways by miR-1897-3p. The top two targets generated by mirBase v.19 were Lass4 and Nucks1 with Target Scores of 80 and 79 respectively [14]–[17]. TargetScan Mouse 6.2 identified Nucks1 as the top target (total context score = −0.53) closely followed by Lass4 (total context score = −0.43) when poorly conserved sites were included in the query [18]–[20]. MicroRNA.org (August 2010 release) ranked Nucks1 as 100 with a mirSVR score of −0.59 [21]–[22]. Pathway analysis was performed on Nucks1 (nuclear casein kinase and cyclin-dependent kinase substrate 1) using Ingenuity Pathway Analysis (IPA, Qiagen, Redwood City, CA). Indeed, downstream targets of Nucks1 are involved in renal injury, inflammation, and apoptosis (Figure 6). Experimentally, Nucks1 gene expression was down-regulated in the injured kidneys at all timepoints and reached significance at 3 and 24 hours (Figure 7). These findings suggest that miR-1897-3p may, in fact, serve as a marker of renal injury and contribute to progression of renal dysfunction following an ischemic event.

## Discussion

We have induced bilateral renal ischemia in mice by clamping the renal pedicle for 27 minutes. The model produced highly reproducible, progressive, concordant elevation of miR-714, miR–1188, miR-1897-3p, miR-877*, and miR-1224 in plasma and kidneys at 3, 6 and 24 hours after acute kidney injury. These dynamics correlated with histologic findings of kidney injury and with increases in concentrations of a standard marker of renal dysfunction (creatinine). Pathway analysis revealed close association between miR-1897-3p and Nucks1 gene expression that, in turn, targets genes linked to renal injury, inflammation and apoptosis.

MicroRNAs are recognized as crucial regulators of gene expression. MicroRNAs are short non-coding RNAs that regulate gene expression by binding to specific mRNA targets and promoting their degradation and/or translational inhibition [5]. Originally, microRNA analysis was focused on the gene regulation within the same cell or tissue [23]. However, after discovering remarkable stability of microRNA in biological fluids, microRNA in blood and urine has become a popular object in a quest for circulating biomarkers of various diseases [24]. In that context, comparison between microRNA profile in the tissue of interest and in circulation is considered a logical step towards building the case for microRNA as a circulating biomarker of tissue pathology. To the best of our knowledge, there is only one publication to date that attempted systematic profiling of renal and circulating microRNA in the rodent models of AKI. Using the rat model, Saikumar et al. reported that miR-21 and -155 were significantly elevated in the kidneys and lowered in plasma as measured at 24, 72 and 120 hours following reperfusion [11]. In our experiments (data not shown) these microRNA species responded only mildly. MiR-21 increased 1.98 fold in the kidney and 2.1 fold in the plasma at 24 hours. MiR-155 was unchanged in the kidneys but was elevated in the plasma 5.7 fold at 6 hours and 4.1 fold at 24 hours. Given the difference of animal species, analytical platform and, most importantly, the time frame of ischemia-reperfusion, discrepancy between our findings and these data are not surprising. Even regarding microRNA changes in the kidneys per se, several papers have shown vastly different microRNA response [10,11,19,20 25,26]. Interestingly, up-regulation of kidney miR-21 expression was confirmed by two independent groups [11], [19], [24]. It is plausible that miR-21 is elevated as a late response to injury.

Moreover, it could reflect the beginning of tissue restoration since up-regulation of miR-21 was recently associated with renal protection induced by delayed ischemic pre-conditioning [13] [27]. This observation could be of particular importance knowing that, as opposed to human kidneys, rodent kidneys are capable of fast regeneration after acute injury [14] [28]. Therefore, microRNA species responding very early after AKI in rodents might be more relevant to the injury and hence have higher probability of human translation as suggested by the data of Lan et al. [9].

Ramachandran et al reported miR-21, miR-200c, miR-423, and miR-4640 being present in the urine of patients with AKI [29]. Similarly, in our study (data not shown), miR-21 was upregulated 2-fold in both the ischemic kidneys and plasma at 24 hrs, and miR-423-3p was upregulated 4-fold in the plasma of animals that had undergone ischemia. However, we found no change in miR-200c in either the kidneys or plasma. Our array did not include miR-4640.

Mechanistic relationships between renal and circulating microRNAs are not well understood. Although we and others [11] have reported correlations between several microRNA species in these two compartments, it is not clear if this subset of circulating microRNAs was solely of renal origin. The whole body responds to renal ischemia-reperfusion injury, and circulating microRNAs could have originated elsewhere. Moreover, even if injured kidneys were the primary source of circulating microRNAs, more studies are needed to identify specific cell type producing them. Nevertheless, given that among multiple microRNAs modulated by ischemia-reperfusion in the kidneys and plasma, only few species were changed in a coordinated manner, it is tempting to speculate that circulating microRNAs were at least partially originating from the kidneys.

Potential mechanism of microRNA transport from cell to plasma is also unclear. Elevation of certain microRNAs in plasma may represent a result of renal cell death associated with leakage of cytoplasmic components. Alternatively, microRNAs can be actively secreted from the surviving cells inside membrane microparticles, exosomes, or in a membrane-free, protein-bound form [15], [16] [30], [31]. Elucidation of these mechanisms might provide better understanding of the nature of direct and inverse relationships between renal and plasma microRNAs. An intriguing possibility exists that circulating microRNAs could play an important role in inter-organ communication rather than being mere bystanders of tissue injury [6].

Regardless of their biogenesis and mechanistic involvement, though, extracellular microRNAs have potential to be utilized as circulating biomarkers of AKI. Ideal biomarkers should be accessible through non-invasive methods, specific to the disease or pathology of interest, should facilitate early detection, be sensitive to change in pathology and be easily translatable from model systems to humans [4]. In the case of AKI, both plasma and urine microRNAs fulfill some of the above criteria while their fitness to other requirements still needs to be addressed. While both plasma and urine microRNAs have shown some clinical promise [7], [8], in the current study we were limited to analysis of circulating plasma microRNAs since the mice do not produce sufficient amount of urine early after severe renal ischemia-reperfusion insult. Taking into consideration that oliguria is one of the main symptoms and diagnostic criteria of AKI in the clinic, this focus on plasma microRNAs could also have translational value.

From the translational perspective, it is critical to evaluate relationships between potential novel biomarkers and established marker(s) of the disease. We have demonstrated strong correlation of plasma miR-714, miR-1188 and miR-1897-3p and a conventional marker, creatinine. As expected, creatinine levels tightly correlated with kidney histopathology. However, we have not found dramatic advantages of circulating microRNAs compared to creatinine in terms of sensitivity. Therefore, in this case, identification of novel circulating microRNAs would not necessarily have potential to improve early clinical diagnosis of AKI. It remains to be seen, however, if these markers have significant advantages regarding their response to drug treatment. While using circulating microRNAs as a pharmacodynamic marker is essentially an uncharted territory, it is reasonable to expect that for compounds with well- defined mechanisms of action, they might offer better sensitivity and specificity. Unlike creatinine that provides an overall measure of kidney function, individual microRNAs are likely to reflect more specific molecular mechanisms of the disease and their modulation by pharmacological treatment.

To our knowledge, this is the first time that miR-1188, miR-1897-3p, and miR-877* have been shown to be involved in ischemic tissue injury. In an analogous model of vascular end-organ damage, miR-714 appears to play a role in the process of vascular calcification in mice [32], suggesting that miR-714 may have a broad role in vascular homeostasis. While Hunsberger et al. also report that miR-1224 is upregulated in response to cerebral ischemia in rats [33], it has yet to be determined whether these microRNAs are causative or merely responding to injury.

Of interest, in the current study, pathway analysis suggested potential mechanistic connection between miR1897-3p, Nucks1 and MME (neprilysin). Specifically, miR1897-3p levels were elevated both in the kidneys and plasma at all time points. MiR1897-3p is supposed to regulate expression of Nucks1 that, in turn, controls expression of neprilysin. Indeed, RT-PCR demonstrated significant changes in Nucks1 gene expression in the kidneys. Admittedly, this alleged connection needs to be tested separately in a more direct mechanistic fashion. However, it generated some hints of translational value. Urine neprilysin has been previously reported as a marker of kidney injury in the clinic [34]. At the same time, neprilysin is an established drug target due to its effects on degradation of natriuretic peptides [35]. Therefore, miR1897-3p holds the promise to serve as a pharmacodynamic marker for neprilysin inhibitors. Similar logic could be applicable to other circulating microRNA species in a context of drug discovery and development as it relates to the compounds with different mechanism of action.

The present study was of a pilot nature, and data interpretation was limited by the lack of mechanistic experiments that would focus on tissue-restricted genetic manipulations with individual microRNAs. However, we believe that systematic profiling of renal and plasma microRNAs in the early stages of experimental AKI provided the first necessary step in advancing circulating microRNAs to the level of promising novel biomarkers.
